# Abnormal Liver Stiffness Assessed Using Transient Elastography (Fibroscan®) in HIV-Infected Patients without HBV/HCV Coinfection Receiving Combined Antiretroviral Treatment

**DOI:** 10.1371/journal.pone.0052720

**Published:** 2013-01-03

**Authors:** Sang Hoon Han, Seung Up Kim, Chang Oh Kim, Su Jin Jeong, Jun Yong Park, Jun Yong Choi, Do Young Kim, Sang Hoon Ahn, Young Goo Song, Kwang-Hyub Han, June Myung Kim

**Affiliations:** 1 Department of Internal Medicine and AIDS Research Institute, Yonsei University College of Medicine, Seoul, Republic of Korea; 2 Department of Internal Medicine and Liver Cirrhosis Clinical Research Center, Yonsei University College of Medicine, Seoul, Republic of Korea; University of Modena & Reggio Emilia, Italy

## Abstract

**Background and Aims:**

Liver stiffness measurement (LSM) using transient elastography (Fibroscan®) can identify individuals with potential underlying liver disease. We evaluated the prevalence of abnormal LSM values as assessed using LSM and its predictors in HIV-infected asymptomatic patients receiving combined antiretroviral treatment (cART) without HBV/HCV coinfection.

**Methods:**

We prospectively recruited 93 patients who had consistently been undergoing cART for more than 12 months at Severance Hospital in Seoul, Republic of Korea, from June to December 2010. LSM values >5.3 kPa were defined as abnormal.

**Results:**

Thirty-nine (41.9%) had abnormal LSM values. On multivariate correlation analysis, the cumulative duration of boosted and unboosted protease inhibitors (PIs) were the independent factors which showed a negative and positive correlation to LSM values, respectively (*β* = –0.234, *P* = 0.023 and *β* = 0.430, *P*<0.001). In multivariate logistic regression analysis, the cumulative exposure duration of boosted-PIs and γ-glutamyltranspeptidase levels were selected as the independent predictors which showed a negative and positive correlation with abnormal LSM values, respectively (odds ratio [OR], 0.941; 95% confidence interval [CI], 0.889–0.997; *P* = 0.039 and OR, 1.032; 95% CI, 1.004–1.060; *P* = 0.023).

**Conclusion:**

The high percentage of HIV-infected asymptomatic patients receiving cART without HBV/HCV coinfection had abnormal LSM values. The cumulative exposure duration of boosted-PIs and γ-GT level were independent predictors which showed a negative and positive correlation with abnormal LSM values, respectively.

## Introduction

Because the life expectancy in HIV-infected individuals has increased continuously since the introduction of effective combined antiretroviral treatment (cART), non-AIDS defined complications, such as cardiovascular disease, osteoporosis, non-AIDS defining malignancies, renal disease, and chronic liver disease, are now being considered more important causes of morbidity and mortality than opportunistic infections (OIs). [1]–[7].

Notably, chronic liver disease has become an important cause of morbidity and mortality in HIV-infected individuals. [2], [7] Although hepatitis B (HBV) or C virus (HCV) coinfection is the main cause of chronic liver damage, alcohol consumption, drugs (especially antiretroviral drugs), and non-alcoholic steatohepatitis can be other causes. [7] Among these, due to long-term use of cART, many HIV-infected patients are facing potential sequelae of cART-induced hepatotoxicity, such as the development of liver fibrosis. [7].

If elevation of liver enzymes is identified during cART at visits of regular follow-up, physicians can modify their treatment strategies. However, these adverse reactions by cART are often subject to physician indifference because 50% of patients are asymptomatic when liver enzymes are raised. [8] Furthermore, HIV-infected patients, even those with no elevation of liver enzymes, are also vulnerable to asymptomatic liver damage. Thus, early detection of asymptomatic liver damage showing normal liver enzyme in HIV-infected patients without HBV or HCV coinfection receiving long-term cART is of paramount importance to preventing the silent progression of liver fibrosis, resulting in advanced fibrosis or cirrhosis.

Recently, noninvasive liver stiffness measurement (LSM) using transient elastography (Fibroscan**®)** has been performed to evaluate the severity of liver fibrosis and to screen the general population for subjects at risks of having underlying liver disease with high reproducibility. [9], [10] Using LSM, one previous study reported prevalence and risk factors for abnormal LSM values in HIV-infected patients without viral hepatitis coinfection. [11] However, the cutoff LSM value used in that study to define HIV-infected patients with abnormal LSM values was adopted from the LSM value for predicting significant fibrosis in HIV/HCV co-infected patients who may already have had a fibrotic background liver due to chronic HCV infection, leading to the cutoff LSM values being elevated. [11] Thus, the overestimated cutoff LSM values in the previous study may have underestimated the prevalence of HIV-infected patients with abnormal LSM values.

In this study, to overcome the confounding effect of coinfection with hepatitis virus, we adopted cutoff LSM values derived from healthy individuals [12] and investigated the prevalence of abnormal LSM values and its predictors in HIV mono-infected asymptomatic patients.

## Materials and Methods

### Study Participants and Design

We prospectively enrolled 125 HIV-infected patients to perform a prospective cross-sectional observational study at Severance Hospital, a 2,000-bed, university-affiliated tertiary teaching hospital in Seoul, Republic of Korea from June to December 2010.

**Figure 1:**
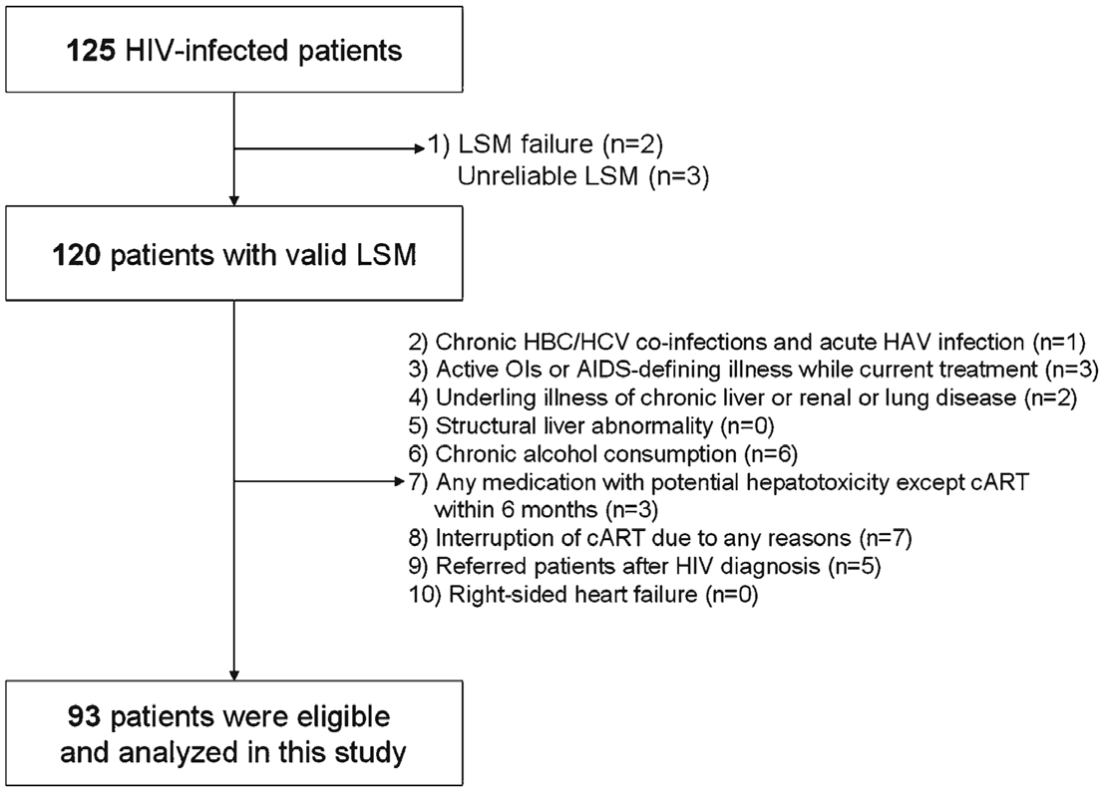
Recruitment flow of study participants for this study. **NOTE.** LSM, liver stiffness measurement; HBV, hepatitis B virus; HCV, hepatitis C virus; HAV, hepatitis A virus; OI, opportunistic infection; AIDS, acquired immunodeficiency syndrome; cART, combined antiretroviral treatment.

To clarify the effects of long-term cART on asymptomatic liver damage, reflected by abnormal LSM values, we included only HIV-infected asymptomatic patients with normal alanine aminotransferase (ALT) and total bilirubin who had continuously received cART more than 12 months and visited regularly at 3-month intervals. We further excluded patients who filled the following exclusion criteria; (1) LSM failure (no valid shots) or unreliable LSM (n = 5, 4.0%) (2) chronic HBV/HCV coinfection or acute hepatitis A virus infection, (3) any active OIs or AIDS-defining illnesses including malignancy while undergoing current treatment, (4) underlying illness or past treatment history of chronic liver or renal or lung disease defined according to the International Classification of Disease, 10th Revision, [13] (5) ultrasonographic evidences of structural liver abnormality, (6) alcohol consumption in excess of 40 g/day for more than 5 years, (7) any medication besides cART with potential hepatotoxicity within 6 months of enrollment (lipid-lowering agents and liver pills or folk medicine including herb *et al*), (8) interruption of cART for any reasons including adverse reactions and poor adherence, (9) patients referred to our hospital after HIV diagnosis, and (10) right-sided heart failure. Taking into account these exclusion criteria, a total of 93 patients were finally recruited (Figure 1).

This study was approved by the local Institutional Review Board of the Clinical Research Institute of Severance Hospital. All participants gave informed consent for their participation in this study.

### Laboratory Tests and Data Collections

In addition to demographic data, CD4+ T lymphocyte count, plasma HIV-RNA viral load (VL), liver function tests, including aspartate aminotransferase (AST), ALT, alkaline phosphatase (ALP), total bilirubin, γ-glutamyl transferase (γ-GT), prothrombin time (PT), and platelet count were collected on the same day as LSM examination. The upper limit of normal (ULN) was set as 40 IU/L for ALT and 1.2 mg/dL for total bilirubin. Thus, patients with abnormal liver-related laboratory findings related to potential underlying liver disease, defined as ALT >40 IU/L and total bilirubin >1.2 mg/dL, were not enrolled in this study [12].

Several covariates have been collected by the retrospective review of the electrical medical record. These included mode of transmission, time since HIV infection diagnosis, history of AIDS defining illness and Centers for Disease Control and Prevention (CDC) category B illness, [14] the nadir CD4+ T lymphocyte count, plasma highest VL value after HIV diagnosis and cART regimens. We reviewed cART in detail, including the total duration of treatment, whether or not antiretroviral drugs were changed, current regimens upon LSM examination, and cumulative exposure duration to each antiretroviral drug after cART initiation.

### Liver Stiffness Measurement and Ultrasonographic Evaluation

LSM using FibroScan® was performed by a single physician blinded to clinical and biochemical data according to the instructions provided by the manufacturer. Details of the technical background and examination procedure have been previously described. [9], [15] The success rate was calculated as the number of valid measurements divided by the total number of measurements. The results were expressed as kilopascals (kPa). Interquartile range (IQR) was defined as an index of intrinsic variability of LSM corresponding to the interval of LSM results containing 50% of the valid measurements between the 25th and 75th percentiles. The median value was considered representative of the elastic modulus of the liver. Only procedures with at least 10 valid measurements, a success rate of at least 60% and an IQR to median value ratio <30% were considered reliable.

Ultrasonographic evaluation was also performed by a single physician blinded to the clinical and biochemical data to exclude any structural or morphological liver abnormalities that could influence LSM values.

### Definition of Abnormal LSM Values

Because HIV mono-infected asymptomatic patients were recruited, we needed cutoff LSM values derived from a healthy population without background chronic liver disease that could raise LSM values and subsequently underestimate the prevalence of patients with abnormal LSM values. Although several previous studies proposed their own normal ranges for LSM values, [16] we adopted 5.3 kPa, which was derived from Korean healthy living-related liver and kidney donors, as the cutoff LSM value for stratifying our study population into HIV-infected asymptomatic patients with normal and abnormal LSM values.

### Statistical Analyses

The data were expressed as the mean ± standard deviation (SD) for continuous variables with normal distribution or median (IQR) without normal distribution and as the number (percent) for all categorical variables. To evaluate the correlation between various covariates and LSM values, we performed univariate Pearson’s or non-parametric (Spearman’s ρ) correlation and multivariate linear regression analysis as the standardized regression coefficients with variables with *P*-value of less than 0.1 upon univariate correlation analysis.

The Student’s independent T-test or Mann-Whitney U-test were used to compare the mean values of continuous variables with normal or skewed distribution between the groups with normal and abnormal LSM values. To analyze the differences in nominal variables between the two groups, we performed a chi-square test or Fisher’s exact test. Finally, multivariate logistic regression analysis was performed to identify the associated clinical factors with abnormal LSM values. In this final model, we included several independent variables with *P*-values of less than 0.1 upon univariate analysis between the two groups, respectively. The optimal cutoff values of the independent variables were selected to maximize the sum of sensitivity and specificity derived from the receiver operating characteristic (ROC) curves and the area under ROC (AUC) analysis.

All *P*-values were two-tailed, and *P*<0.05 was considered to be statistically significant. We used SPSS 18.0 software (SPSS Inc., Chicago, IL, USA) to perform all statistical analysis.

## Results

### The Baseline Characteristics and Antiretroviral Drug Regimens of Study Participants upon LSM Examination

**Figure 2:**
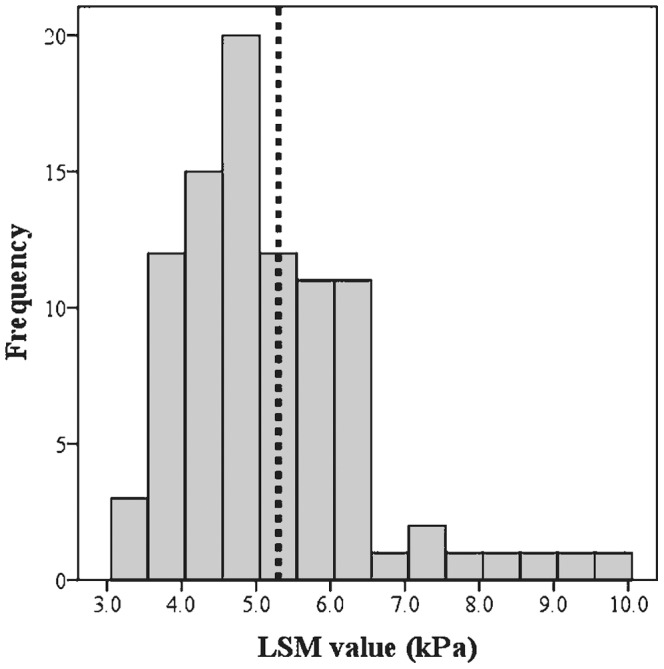
Distribution of LSM values among all study participants. **NOTE.** The dotted line indicates the cutoff value for abnormal LSM (5.3 kPa). LSM, liver stiffness measurement; kPa, kilopascal.

The baseline characteristics and antiretroviral drug regimens of the study participants upon LSM examination are summarized in Table 1. The minimum and maximum LSM values of total patients were 3.3 and 10.0 kPa (Figure 2). The mean value of nadir CD4+ T lymphocyte counts was 150 cells/mm^3^ and the highest plasma HIV-RNA VL was log 5.0 copies/mL.

**Table 1:** Baseline characteristics and antiretroviral drug regimens of study participants upon LSM examination (n = 93).

Variables	Values
Age, years	42.9±10.3
Male gender	88 (94.6)
Mode of transmission	
Heterosexual/homosexual/not clarified	40 (43.0)/24 (25.8)/29 (31.2)
Change of antiretroviral drug^a^	47 (50.5)
Time since HIV infection diagnosis, months	78.9±66.9
Total cART duration, months	50.4±37.8
Past history of AIDS defining illness and CDC category B illness	48 (51.6)
CD4+ T lymphocyte counts, cells/mm^3^	502.9±245.5
Plasma HIV-RNA viral load (<20 copies/mL)	76 (81.7)
LSM value, kPa	4.9 (4.3–5.9)
Body mass index, kg/m^2^	22.4±3.0
Liver function tests	
Alanine aminotransferase, IU/L	19.4±5.7
Alkaline phosphatase, IU/L	62.2±22.8
Total bilirubin, mg/dL	0.71±0.16
γ-glutamyltranspeptidase, IU/L	49.8±47.3
Prothrombin time, INR	0.92±0.06
Platelet count, X 10^3^/mm^3^	260.9±229.1
Antiretroviral drugs at LSM examination	
NRTIs	
Zidovudine/didanosine/stavudine/abacavir	44 (47.3)/28 (30.1)/4 (4.3)/22 (23.7)
NNRTI (all efavirenz)	39 (41.9)
PIs	53 (57.0)

**NOTE.** Data are expressed as mean ± SD, number (percent), or median (interquartile range). LSM, liver stiffness measurement; cART, combined antiretroviral treatment; AIDS, Adult immunodeficiency syndrome; CDC, Centers for Disease Control and Prevention; kPa, kilopascal; INR, international normalized ratio; NRTIs, nucleoside analogue reverse transcriptase inhibitors; NNRTIs, non-nucleoside analogue reverse transcriptase inhibitors; PIs, protease inhibitors. ^a^A change to co-formulated drugs with the same components was not considered to be a change in antiretroviral drugs.

The mean value of cumulative exposure duration of each nucleoside analogue reverse transcriptase inhibitor (NRTI) drug until LSM examination was zidovudine (AZT) 23.2, didanosine (ddI) 14.0, stavudine (d4T) 7.6, and abacavir (ABC) 8.4 months, respectively. In addition, the mean values of cumulative exposure duration of efavirenz (EFV) and low-dose ritonavir (RTV) boosted lopinavir (LPV/r) were 19.3 and 18.9 months, respectively.

### Correlation between LSM Values and Other Variables

On univariate analysis, the cumulative exposure duration of unboosted PIs had a significant positive correlation with LSM value (*r* = 0.401, *P = *0.025), whereas CD4+ T lymphocyte counts and boosted PIs showed borderline correlations with LSM values, respectively (*r* = –0.186, *P = *0.074 and *r* = –0.241, *P = *0.092). However, on subsequent multivariate linear regression analysis, the cumulative exposure duration of boosted and unboosted PIs were selected as independent factors that had a significant negative and positive correlation with LSM value, respectively (*β* = –0.234, *P = *0.023 and *β = *0430, *P*<0.001) (Table 2).

**Table 2:** Correlation between LSM values and other variables.

Independent variables	Univariate^a^	Multivariate
	*r* (*P-*value)	*β* (*P-*value)
Age, years	0.002 (0.984)	–
Time since HIV infection diagnosis, months	−0.086 (0.412)	–
Total duration of cART, months	−0.011 (0.917)	–
CD4+ T lymphocyte counts, cells/mm^3^		
At LSM examination	−0.186 (0.074)	−0.755 (0.452)
Nadir	−0.039 (0.714)	–
Plasma HIV-RNA viral load, copies/mL		
At LSM examination	−0.017 (0.868)	–
Log_10_(highest value after the HIV diagnosis)	0.090 (0.402)	–
Body mass index, kg/m^2^	−0.025 (0.819)	–
Alanine aminotransferase (IU/L)	0.120 (0.251)	–
Alkaline phosphatase (IU/L)	0.143 (0.170)	–
Total bilirubin (mg/dL)	−0.037 (0.725)	–
γ-glutamyltranspeptidase (IU/L)	0.119 (0.525)	–
Prothrombin time, INR	0.181 (0.241)	–
Platelet count, X 10^3^/mm^3^	−0.112 (0.287)	–
Cumulative exposure duration of antiretroviral drugs, months^b^		
NRTIs		
Zidovudine (n = 68)	0.127 (0.301)	―
Stavudine (n = 25)	−0.247 (0.234)^c^	―
Didanosine (n = 39)	0.117 (0.477)	―
Abacavir (n = 23)	−0.059 (0.788)^c^	―
NNRTIs (n = 54)	0.230 (0.095)	―
PIs (n = 58)	0.043 (0.747)	
Boosted (n = 50)	−0.241(0.092)	−0.234 (0.023)
Unboosted (n = 31)	0.401 (0.025)	0.430 (<0.001)

**NOTE.** ^a^Pearson’s correlation coefficient, ^b^Correlation analyses in only patients who has ever been received each antiretroviral drug, ^c^Spearman’s ρ. LSM, liver stiffness measurement; cART, combined antiretroviral treatment; INR, international normalized ratio; NRTIs, nucleoside analogue reverse transcriptase inhibitors; NNRTIs, non-nucleoside analogue reverse transcriptase inhibitors; PIs, protease inhibitors.

### Comparison between Patients with Normal and Abnormal LSM Values

Thirty nine subjects (41.9% of the study population) showed abnormal LSM values (Table 3), and these patients had a significantly shorter time period from HIV infection diagnosis than those with normal LSM values (*P* = 0.049). Although statistical significance was borderline, patients with abnormal LSM values had higher nadir CD4+ T lymphocyte counts (*P* = 0.082), lower total bilirubin (*P* = 0.050), higher γ-GT (*P* = 0.086), lower PT (*P* = 0.099), and absence of exposed history of boosted-PIs (*P* = 0.094) (same order with table) than those with normal LSM values upon univariate analysis (Table 3). Any significant differences were not revealed in comparisons of current antiretroviral regimens at the LSM examination between patients with normal and abnormal LSM values (data not shown). When the cumulative exposure duration of each antiretroviral drug was compared between patients with normal and abnormal LSM values, only the exposure duration of boosted PI was significantly different (mean 26.5 *vs*. 14.0 months; *P* = 0.021).

**Table 3:** Comparisons of baseline characteristics and cumulative exposure durations of antiretroviral drugs between patients with normal and abnormal LSM values.

Variables	Patients with normal LSMvalues (n = 54, 58.1%)	Patients with abnormal LSMvalues (n = 39, 41.9%)	*P-*value
Age, years	42.1±9.8	43.9±11.1	0.421^a^
Gender, male	52 (96.3)	36 (92.3)	0.646^b^
Time period since HIV infection diagnosis, months	90.5±80.9	62.9±35.5	0.049^a^
Total duration of cART, months	62.1±36.7	50.5±30.3	0.108^a^
CD4+ T lymphocyte counts, cells/mm^3^			
At LSM examination	528.0±239.7	468.0±252.3	0.247^a^
Nadir	132.7±99.3	173.6±120.6	0.082^a^
Plasma HIV-RNA viral load, copies/mL			
Undetectable range^e^ at LSM examination, yes, n (%)	46 (85.2)	30 (76.9)	0.416^c^
Log_10_(highest value after the HIV diagnosis)	5.0±1.0	5.0±0.8	0.832^a^
Body mass index, kg/m^2^	22.5±3.0	22.4±2.9	0.860^a^
Alanine aminotransferase, IU/L	22.9±12.3	26.6±17.4	0.238^a^
Alkaline phosphatase, mg/dL	61.8±26.3	62.7±17.1	0.863^a^
Total bilirubin, mg/dL	1.0±1.0	0.7±0.5	0.050^a^
γ-glutamyltranspeptidase, IU/L	36.4±29.5	74.2±63.7	0.086^a^
Prothrombin time, INR	0.93±0.06	0.90±0.06	0.099^a^
Platelet count, X 10^3^/mm^3^	287±294	225±61	0.195^a^
Exposed history of antiretroviral drugs, yes, n (%)			
NNRTIs	33 (61.1)	21 (53.8)	0.484^c^
PIs	36 (66.7)	22 (56.4)	0.314^c^
Boosted	33 (61.1)	17 (43.6)	0.094^c^
Unboosted	18 (33.3)	13 (33.3)	1.000^c^
NRTI backbone			
Zidovudine	39 (72.2)	29 (74.4)	0.819^c^
Stavudine	17 (31.5)	8 (20.5)	0.239^c^
Didanosine	22 (40.7)	17 (43.6)	0.784^c^
Abacavir	16 (29.6)	7 (17.9)	0.198^c^

**NOTE.** Data are expressed as mean ± SD or number (percent). ^a^Independent sample two T-test, ^b^Fisher’s exact test, ^c^Chi-square test, and ^d^Mann-Whitney U-test were used.^ e^The undetectable range was defined as fewer than 20 copies/mL. LSM, liver stiffness measurement; cART, combined antiretroviral treatment; INR, international normalized ratio; NNRTI, non-nucleoside analogue reverse transcriptase inhibitor; cART, combined antiretroviral treatment; PI, protease inhibitors; NRTI, nucleoside analogue reverse transcriptase inhibitor.

### Independent Predictors of Abnormal LSM Values

**Figure 3:**
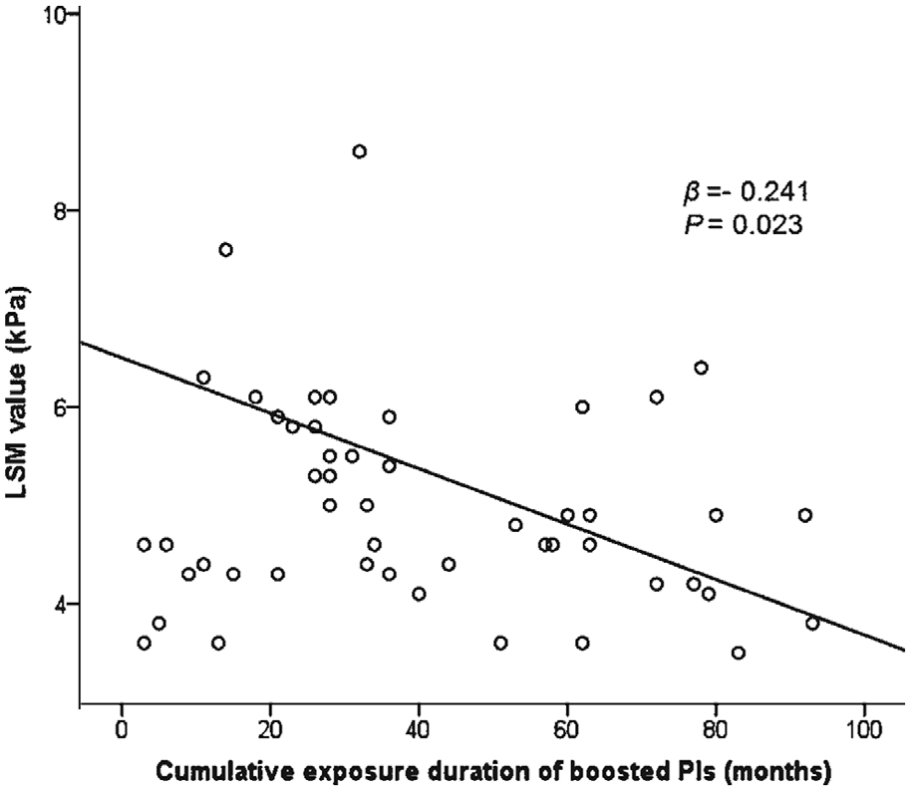
Scatter plot showing the correlation between antiretroviral treatment and LSM value in patients who have been exposed to boosted PIs (n = 50) among study participants (*β* = −0.234, *P* = 0.023).

On multivariate logistic regression analysis, the cumulative exposure duration of boosted PIs and γ-GT level were selected as independent predictors which showed a negative and positive correlation with abnormal LSM values, respectively (odds ratio [OR], 0.941; 95% confidence interval [CI], 0.889–0.997; *P* = 0.039 and OR, 1.032; 95% CI, 1.004–1.060; *P* = 0.023) (Table 4). The significant correlation between the cumulative exposure duration of boosted PIs and LSM values is described in Figure 3.

**Table 4:** Multivariate logistic regression analysis to identify the independent predictors of abnormal LSM values.

Independent variables	OR	95% CI	*P*-value
Cumulative exposure duration of boosted-PIs	0.941	0.889–0.997	0.039
γ-glutamyltranspeptidase, IU/L	1.032	1.004–1.060	0.023

Included variables in this final model: Cumulative exposure duration of boosted-PIs, γ-glutamyltranspeptidase, total bilirubin, time period since HIV infection diagnosis, and prothrombin time. LSM, liver stiffness measurement; OR, odds ratio; CI, confidence interval; PIs, protease inhibitors.

### Optimal Cutoff Values for Independent Predictors of Abnormal LSM Values

The optimal cutoff values of the cumulative exposure duration for boosted-PIs and γ-GT were calculated as 33 months (*P* = 0.041; area under the ROC curve [AUROC] = 0.619; 95% CI, 0.506–0.733; sensitivity, 38.9%; specificity, 87.2%; PPV, 80.8%, and negative predictive value [NPV], 50.7%) and 32 IU/L (*P* = 0.010; AUROC = 0.782; 95% CI, 0.606–0.958; sensitivity, 42.6%; specificity, 38.5%; PPV, 48.9%, and NPV, 32.6%).

**Figure 4:**
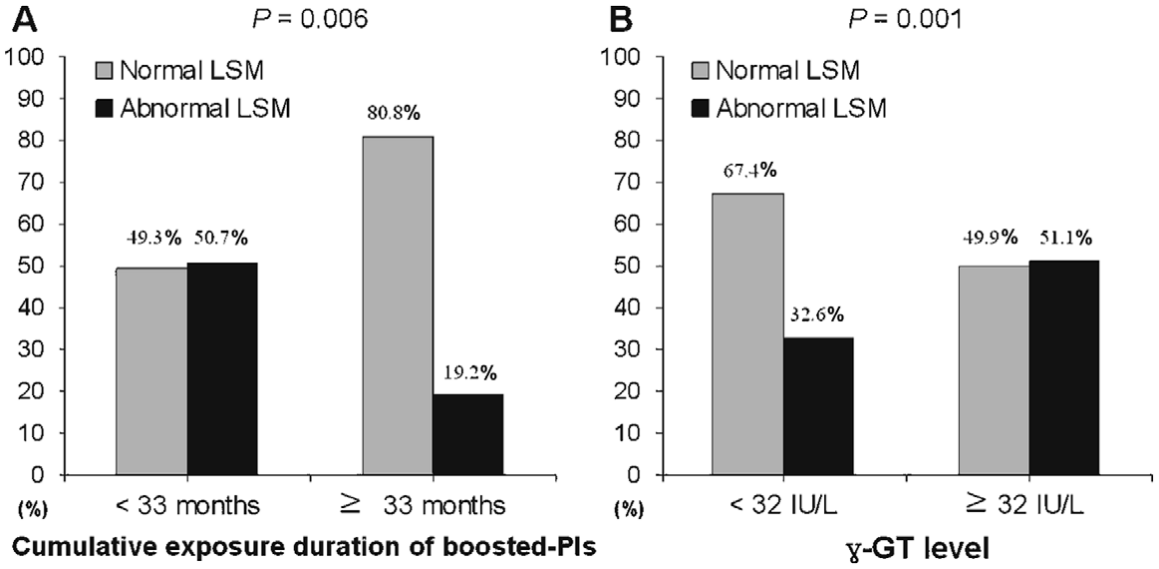
Percentage of patients with abnormal LSM values according to cutoff values of the cumulative exposure duration of boosted-PIs (a) and γ-GT level (b).

Abnormal LSM values were observed in 34 of 67 (50.7%) patients with cumulative exposure duration of boosted-PIs of <33 months and 5 of 26 (19.2%) patients with cumulative exposure duration of boosted-PIs of ≥33 months (OR, 0.61; 95% CI, 0.45–0.83; *P* = 0.006) (Figure 4a). Similarly, abnormal LSM values were observed in 15 of 46 (32.6%) patients with γ-GT level <32 IU/L and 24 of 47 (51.1%) patients with γ-GT level ≥32 IU/L (OR, 2.49; 95% CI, 1.30–4.75; *P* = 0.001) (Figure 4b).

## Discussion

Recently, there has been increasing concern regarding cART-induced hepatotoxicity. [7] cART may cause liver damage via a variety of mechanisms such as mitochondrial toxicity, drug hypersensitivity reactions, idiosyncratic hepatotoxicity, immune reconstitution syndrome and hepatic steatosis *et al.* [17].

If HIV-infected patients have no subjective symptoms and show normal ALT range and total bilirubin levels similar to our study population, potential liver damage seems to be difficult to recognize in clinical practice. However, it has been revealed that a significant proportion of HIV-infected patients receiving cART without HBV/HCV coinfection showed abnormal LSM values, indicating potential asymptomatic liver damage. To our knowledge, this is the first cross-sectional study to evaluate the prevalence of abnormal LSM values in HIV-infected asymptomatic patients receiving long-term cART without HBV/HCV co-infection, assessed using cutoff LSM values derived from healthy population.

Although several studies have concluded that HIV-infected patients with HBV or HCV coinfection have significantly more advanced liver fibrosis than those without co-infection with the aid of LSM, [18]–[20] the assessment of asymptomatic liver damage using LSM in HIV mono-infected patients receiving long-term cART has been rarely addressed. To date, one study evaluated the prevalence and risk factors for abnormal LSM values without coinfection using the cutoff LSM value of 7.2 kPa, concluding that the prevalence of liver damage was 11.2% and that long-term exposure to ddI is a major risk factor for abnormal LSM values. [11] However, because 7.2 kPa might have been overestimated due to underlying liver fibrosis caused by chronic HCV infection, it might have reduced the prevalence of abnormal LSM values in HIV mono-infected asymptomatic patients. Another recent study by Stabinski et al. which used a cutoff value of 9.3 kPa for diagnosing significant fibrosis, not for asymptomatic liver damage, concluded that the burden of liver fibrosis among HIV-infected rural Ugandans is high and that the prevalence of significant fibrosis in HIV-infected individuals was significantly higher than non-HIV-infected individuals (17% *vs*. 11%, *P* = 0.008) [21]. However, patients with mild asymptomatic liver damage might have been improperly stratified into the normal group.

Considering that early detection of asymptomatic liver damage in HIV mono-infected patients receiving long-term cART and subsequent modification of treatment strategies to prevent additional liver damage is of paramount important and that confounding liver damage by other viruses should be excluded from the evaluation of HIV mono-infected patients, the LSM cutoff value used to identify patients with abnormal LSM values in previous studies does not seem optimal. Indeed, the prevalence of patients with abnormal LSM values in our study (41.9%) using relatively lower cutoff LSM values derived from a healthy population was significantly higher than those of two previous studies (11.2% and 11%). [11], [21] These results might also support the idea that the cutoff LSM values in previous studies might have been biased due to the confounding effect of liver damage from the hepatitis virus and the commitment to predict more than significant fibrosis.

In this study, the cumulative exposure durations of boosted-PIs and the γ-GT level were selected as significant predictors which showed a negative and positive correlation with LSM values, respectively. The use of low-dose RTV (100 mg/day) showed the protective effects for abnormal LSM values in our study. PIs have been frequently associated with the development of various metabolic complications. [22] These have been associated with liver damage by non-alcoholic fatty liver disease. [23] Low-dose RTV is a strong hepatic cytochrome P450 3A inhibitor that helps boost the pharmacokinetics of PI drugs and seems to be well tolerated. [24] Two previous studies reported that cART with LPV/r showed a low incidence of hepatotoxicity. [25], [26] The inhibitory effects of low-dose RTV on the metabolism of PIs in the liver might result in a protective role of boosted PIs on abnormal LSM values. However, the precise mechanism for role of boosted PIs on abnormal LSM values is unknown at present. Therefore, future laboratory and clinical research is warranted to clarify this result.

In addition, γ-GT level was also identified as an independent predictor of abnormal LSM values. Among various etiologies for elevated γ-GT, alcohol consumption can be suspected first, although patients with heavy alcohol consumption were excluded from our study. Thus, when isolated γ-GT elevation was noted in HIV-infected patients, detailed history-taking regarding alcohol consumption and abstinence from alcohol should first be attempted. The calculated optimal cutoff value of γ-GT (32 IU/L) was significantly lower than ULN of γ-GT (54 IU/L) [12] indicating that abnormal LSM values can be identified even in subjects with normal γ-GT level. However, because the usefulness of elevated γ-GT level is limited due to its lack of specificity in spite of high sensitivity in detecting hepatobiliary disease, the clinical implications of using γ-GT to detect abnormal LSM values in HIV-patients should be further investigated.

This study has the some limitations. First, liver biopsy was not performed. Thus, we could not confirm that patients with normal LSM values had histologically normal livers. We were thus unable to exclude the potential presence of OIs in the liver such as cytomegalovirus and herpes simplex virus, due to the absence of biopsy data. However, liver biopsy in asymptomatic patients with normal ALT and total bilirubin level is not ethical in clinical practice and the risk of OIs could have been ignored due to the high mean CD4+ T lymphocyte count of 503 cells/mm^3^ upon LSM examination. Second, the majority of our study participants were male. Because LSM values might differ between genders, [27] larger scale studies including a sufficient number of female HIV-infected patients should be conducted in the future. Third, because this was a cross-sectional study, a long-term follow-up longitudinal study should reveal whether we should modify treatment strategy for patients showing abnormal LSM values to improve treatment outcomes and whether treatment outcomes are really poorer in HIV mono-infected patients with abnormal LSM values than in those with normal LSM values.

In conclusion, we identified a high prevalence of abnormal LSM values in HIV-infected asymptomatic patients receiving cART without HBV/HCV coinfection. We also found that the cumulative exposure of boosted-PIs and γ-GT level were independent predictors which showed a negative and positive correlation with abnormal LSM values, respectively.
